# Annotation of expressed sequence tags for the East African cichlid fish *Astatotilapia burtoni *and evolutionary analyses of cichlid ORFs

**DOI:** 10.1186/1471-2164-9-96

**Published:** 2008-02-25

**Authors:** Walter Salzburger, Susan CP Renn, Dirk Steinke, Ingo Braasch, Hans A Hofmann, Axel Meyer

**Affiliations:** 1 Lehrstuhl für Zoologie und Evolutionsbiologie, Department of Biology, University of Konstanz, 78467 Konstanz, Germany; 2 Zoological Institute, University of Basel, 4051, Switzerland; 3 Department of Biology, Reed College, Portland, Oregon 97202, USA; 4 Guelph Centre for DNA Barcoding, Biodiversity Institute of Ontario, University of Guelph, Guelph, Ontario N1G 2W1, Canada; 5 Physiological Chemistry I, Biozentrum, University of Würzburg, 97074 Würzburg, Germany; 6 Section of Integrative Biology, University of Texas at Austin, Austin, Texas 78712, USA

## Abstract

**Background:**

The cichlid fishes in general, and the exceptionally diverse East African haplochromine cichlids in particular, are famous examples of adaptive radiation and explosive speciation. Here we report the collection and annotation of more than 12,000 expressed sequence tags (ESTs) generated from three different cDNA libraries obtained from the East African haplochromine cichlid species *Astatotilapia burtoni *and *Metriaclima zebra*.

**Results:**

We first annotated more than 12,000 newly generated cichlid ESTs using the Gene Ontology classification system. For evolutionary analyses, we combined these ESTs with all available sequence data for haplochromine cichlids, which resulted in a total of more than 45,000 ESTs. The ESTs represent a broad range of molecular functions and biological processes. We compared the haplochromine ESTs to sequence data from those available for other fish model systems such as pufferfish (*Takifugu rubripes *and *Tetraodon nigroviridis*), trout, and zebrafish. We characterized genes that show a faster or slower rate of base substitutions in haplochromine cichlids compared to other fish species, as this is indicative of a relaxed or reinforced selection regime. Four of these genes showed the signature of positive selection as revealed by calculating K_a_/K_s _ratios.

**Conclusion:**

About 22% of the surveyed ESTs were found to have cichlid specific rate differences suggesting that these genes might play a role in lineage specific characteristics of cichlids. We also conclude that the four genes with a K_a_/K_s _ratio greater than one appear as good candidate genes for further work on the genetic basis of evolutionary success of haplochromine cichlid fishes.

## Background

The exceptionally diverse species flocks of cichlid fishes in the East African Great Lakes Tanganyika, Malawi and Victoria are prime examples for adaptive radiations and explosive speciation [1-3]. More than 2,000 cichlid species have evolved in the last few million years in the rivers and lakes of East Africa [1,4-6]. Together with an additional ~1,000 species that are found in other parts of Africa, in South and Central America, in Madagascar, and in India, the family Cichlidae represents one of the most species-rich families of vertebrates. In addition to their unparalleled species-richness, cichlids are famous for their ecological, morphological and behavioral diversity [1,2,7], for their propensity for rapid speciation [5], for their capacity for sympatric speciation [8,9], and for the formation of parallel characters in independently evolved species flocks [10-12]. For these reasons, the cichlid fishes are an excellent model system to study basic dynamics of evolution, adaptation and speciation. However, while the phylogenetic relationships between the main cichlid lineages are largely established and some of the cichlids' evolutionary innovations have been identified [1,2,4,7,13], little is known about the genomic and transcriptional basis of the evolutionary success of the cichlids.

The cichlid model system provides many advantages for evolutionary genomic research. The hundreds of closely related yet morphologically diverse species in East Africa's cichlid species flocks are even more powerful than a 'mutagenic screen' (to which these species assemblages have been compared [1,12]) in that they represent combinations of alleles that confer a selective advantage under various ecological pressures. Because of the possibility to produce viable crosses between different cichlid species in the lab [14], these alleles can be tied to particular phenotypic traits by means of classical genetic experiments [15-18]. The close relatedness of the different species allows the design of primer sets for the amplification of particular genomic DNA regions such as candidate gene loci, microsatellites, or SNPs, which are applicable to a wide range of species [17,19-21]. The same is true for expression profiling with cDNA microarrays that, once developed for one species, can be used for any East African cichlid species [22].

A variety of genomic resources have already been established for East African cichlid species. Genetic maps are available for the Nile tilapia *Oreochromis niloticus *[23,24] and the Lake Malawi species *Metriaclima zebra *[17]. BAC libraries have been constructed for *O. niloticus *[25] and *M. zebra *(available at the Hubbard Center for Genome Studies), for the Lake Victoria haplochromine *Paralabidochromis chilotes *[26] and for *Astatotilapia burtoni *from Lake Tanganyika and surrounding rivers [27]. cDNA microarrays are available for *A. burtoni *[22] and for Lake Victoria haplochromines [28,29]. Also, EST sequencing projects have been initiated [30], and a BLAST server for cichlid resources has been established [31]. Recently, the National Institute of Health (NIH) has committed to sequencing four cichlid genomes. A detailed description of genomic resources developed for cichlid fishes is available at [32].

Expressed sequence tags (ESTs) derived from the partial sequencing of cDNA clones provide an economical approach to identify large numbers of genes that can be used for comparative genomic and gene expression studies as well as for the detection of splice variants [33,34]. Furthermore, EST projects facilitate genome annotation and are therefore often applied in addition to genome sequencing projects. Due to the large amount of data available in public databases, ESTs emerge as important resources for comparative genome-wide surveys both among closely and more distantly related taxa [35,36]. A series of software applications have been developed to date to perform such EST-based analyses [37-39]. Since ESTs reflect the coding portions of a genome, they can also be used to test for different evolutionary rates in particular genes when comparing different lineages, and to detect genes that have undergone positive selection [35]. It is generally assumed that genes with a statistically significant increase in substitution rates have experienced relaxed functional constraints, while genes, which have not undergone accelerated substitution rates, have experienced purifying selection and, thus, could not accumulate substitutions at random. Positive Darwinian selection, on the other hand, is a phenomenon where selective pressure is favoring change. Natural selection is commonly thought of as a process of editing genetic change so that only a small number of mutational events are retained in natural populations. Under positive selection, the retention of mutations is much closer to the rate at which mutations occur.

Here we report the collection and annotation of more than 12,000 ESTs generated from two different cDNA libraries obtained from the East African cichlid species *Astatotilapia burtoni*, as well as a smaller cDNA library from the Lake Malawi species *Metriaclima zebra. Astatotilapia burtoni *has long been used as a model system to study cichlid spawning behavior [7,40,41], social interactions [41-44], neural and behavioral plasticity [45,46], endocrinology [47], the visual system [48], as well as cichlid development and embryogenesis [49]. In addition, the phylogenetic position of *A. burtoni *makes this species an ideal model system for comparative genomic research [27]. *Astatotilapia burtoni*, which belongs to the most species-rich lineage of cichlids, the haplochromines, was shown to be a sister group to both the Lake Victoria region superflock (~600 species) and the species flock of Lake Malawi (~1,000 species) [4,5,50,51]. Three highly specialized haplochromine species from two species assemblages, *Paralabidochromis chilotes *and *Ptyochromis sp. *"redtail sheller" from Lake Victoria and *Metriaclima zebra *from Lake Malawi, have already been established as genomic models [16,26,28,30]. Important insight into cichlid (genome) evolution will be afforded by the comparison of their genomes to that of *A. burtoni*, which has a more generalist life style and is likely to resemble the ancestral lineage that seeded the cichlid adaptive radiations in these two lakes [4,7].

For EST sequencing, we utilized a cDNA library from *A. burtoni *brain tissue ('*brain*') that was used for the construction of a cDNA microarray [22] and a newly generated normalized cDNA library constructed from different *A. burtoni *tissues at different developmental stages ('*pinky*'). We annotated the ESTs on the basis of similarity searches with BLAST and using the structured vocabulary provided by the Gene Ontology Consortium [52], based on molecular studies of gene function in various model organisms [53]. For evolutionary analyses, we combined our newly generated ESTs with all available sequence data for haplochromine cichlids [30] and a previously constructed library from skin tissue of the Lake Malawi species *Metriaclima zebra *(W. Salzburger, H. A. Hofmann & A. Meyer; unpublished data), which resulted in a total of more than 45,000 ESTs. We then compared the haplochromine ESTs to sequence data from two pufferfish species (*Takifugu rubripes *and *Tetraodon nigroviridis*), trout, and zebrafish, and identified those ESTs with cichlid specific differences in evolutionary rates with EverEST [37].

## Results

The 14,592 initial sequences were trimmed of vector and low-quality sequences and filtered for minimum length (200 bp cut-off), identifying 12,070 high-quality ESTs (Table 1). More than 11,000 of these ESTs (from 13,056 initial sequences) are derived from two different *Astatotilapia burtoni *cDNA libraries – one made from brain tissue ('*brain*'), the other one from different tissues ('*pinky*') including brain, muscle, skin and fin. The overall quality as measured by sequencing success rate and read-length was better in the '*pinky*' library. Also, there was much less redundancy in the '*pinky*' library (16% *versus *30%), which might be the consequence of the normalization step applied to this library or the use of different source tissues.

**Table 1:** Expressed sequence tag (EST) summary

Total sequences	13,056
High quality sequences	12,070 (between 200 and 1,564 bp)
Brain library (*A. burtoni*) ('brain')	4,570
Mixed tissue library (*A. burtoni*) ('pinky')	6,541
Skin library (*P. zebra*)	959

**Figure 1:**
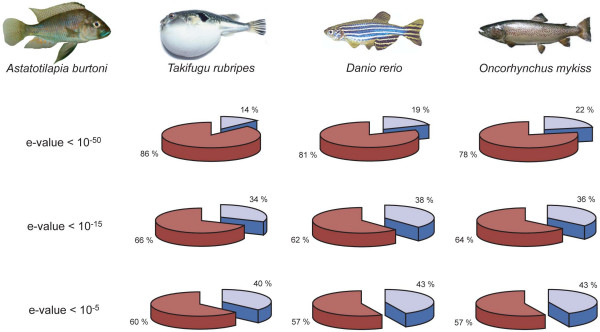
**The proportion of assembled haplochromine cichlid sequences with and without BLAST matches compared to three databases (*Takifugu rubripes*, *Danio rerio*, and *Oncorhynchus mykiss*)**. The pie charts indicate the relative number of BLAST hits (blue) *versus *the percentage fraction, for which no BLAST hit was retrieved (red) for three different e-values (< 10^-50^, < 10^-15^, and <10^-5^, respectively).

A total of 8,636 *A. burtoni *sequences assembled into EST contigs have an open reading frame (ORF) of at least 400 bp. Of these, 1,219 (14%) had matches in the *Takifugu *database and 7,417 (86%) had no matches when an expected value threshold (e-value) of < 1 × 10^-50 ^was used. 2,902 (34%) had matches in the *Takifugu *database with an expected value threshold of < 1 × 10^-15 ^and 3,460 (40%) had matches with an expected value of < 1 × 10^-5^. Similar proportions were retrieved with other databases (Fig. 1).

Among the 8,363 *A. burtoni *assembled sequences, 2,977 could be annotated according to Gene Ontology (GO) terms. Additional files 1, 2 and 3 use the generic GO slim subset of terms ([54]; Generic GO slim; Mundodi and Ireland; downloaded 04/06/2007) that have been developed to provide a useful summary of GO annotation for comparison of genomes, microarrays, or cDNA collections when a broad overview of the ontology content is required. 2,692 ESTs could be assigned to genes listed in the molecular function ontology, 2,532 to genes listed in the biological process ontology, and 2,293 to genes listed in the cellular components ontology, when using an e-value of < 1 × 10^-12^. Additional files 4, 5, and 6 provide more detail of the specific fine-grained terms. Because a single *A. burtoni *assembled sequence may be annotated in all three ontologies and according to multiple ontology terms, a total of 27,451 annotations have been applied (10,926 among biological process, 9,414 among molecular function, and 7,111 among cellular component).

For the comparative evolutionary analyses, we combined our newly generated ESTs with previously published data from *Paralabidochromis chilotes *and *P. sp. *"redtail sheller" [30] and about 1,000 sequences obtained from a *Metriaclima zebra *skin cDNA library (W. Salzburger, H. A. Hofmann & A. Meyer; unpublished data). When using this set of haplochromine cichlid ESTs as reference, we identified 759 open reading frames that are present in all six databases used for comparative analyses (haplochromine cichlids, *Danio rerio*, *Homo sapiens*, *Oncorhynchus mykiss*, *Takifugu rubripes*, and *Tetraodon nigroviridis*).

**Figure 2:**
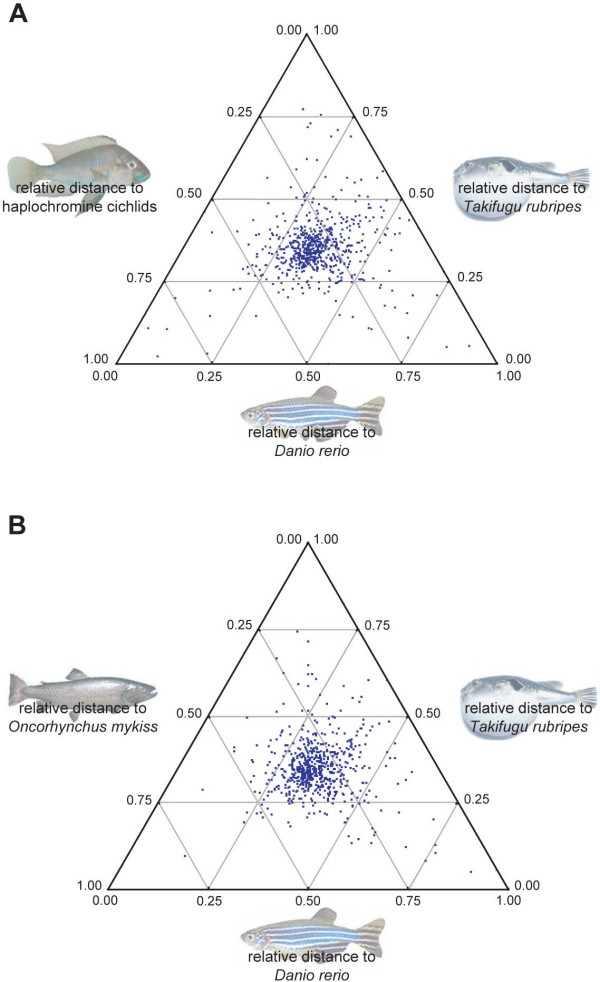
**Ternary representation of relative distances of ORFs of three fish species compared to their human orthologs**. (*a*) Haplochromine cichlid, *Danio rerio*, and *Takifugu rubripes*, (*b*) *Danio rerio*, *Oncorhynchus mykiss*, and *Takifugu rubripes*. Each dot represents a single ORF, the position of the dot within the ternary diagram indicates the relative distance of this ORF in each of the three fish species compared to the orthologous ORF in human. We were interested in identifying those ORFs that show a faster or slower rate of molecular evolution in the haplochromine cichlids.

In order to identify sequences that evolve significantly more rapidly or more slowly in the haplochromine cichlid, we applied the triangle method implemented in EverEST [37] to calculate the p-distance for each of these 759 ORFs in all fish species relative to the human ortholog. There were 22 cases in which more than one haplochromine sequence was found. In these cases, we used the longest sequence for further analyses. The relative p-distances for three fish species were then mapped in ternary diagrams. An example of such a ternary diagram is shown in Fig. 2a, in this case showing the relative p-distances of cichlid, *Takifugu rubripe*s, and *Danio rerio *amino acid sequences with respect to the homologous *Homo sapiens *genes. Figure 2b depicts a diagram with *Oncorhynchus mykiss *amino acid sequence divergence instead of haplochromine cichlid. The ternary diagrams show that in all combinations most genes are clustered around the center of the respective triangle, which indicates that, in general, the p-distances relative to the human outgroup are similar in all fish species.

When compared to the green-spotted pufferfish (*Tetraodon nigroviridis*) and fugu (*Takifugu rubripes*) (always with human as outgroup), 49 gene fragments appeared to have a significantly faster rate of evolution in haplochromine cichlids, and 213 had a slower rate. In the comparison including zebrafish and fugu, 52 genes were found to have evolved faster and 185 genes slower in cichlids. When trout and zebrafish were used, 69 genes were faster and 139 genes evolved slower. In a comparison including trout and fugu, 68 genes appeared to have a faster rate in haplochromines, and 132 had a slower rate. In total 69 genes were found to have evolved faster, and 213 genes appeared to have evolved with a significantly slower mutation rate in haplochromines compared to other fish species. Altogether, about 22% of the surveyed ESTs were found to have haplochromine specific rate differences in at least one of the comparisons suggesting that these genes might play a role in lineage specific features of haplochromine cichlids. A set of 170 cichlid genes appeared in all comparisons. Forty-eight cichlid genes were found to have a higher rate of amino-acid substitution compared to the other fish species included in this study, while 122 cichlid genes were found to have a slower rate. Cichlid sequences that match *Danio rerio*, *Takifugu rubripes*, *Tetraodon nigroviridis*, and *Oncorhynchus mykiss *genes and have a significantly higher or lower p-distance compared to the other fish genes relative to the human outgroup are listed in Additional files 7 and 8, respectively.

**Figure 3:**
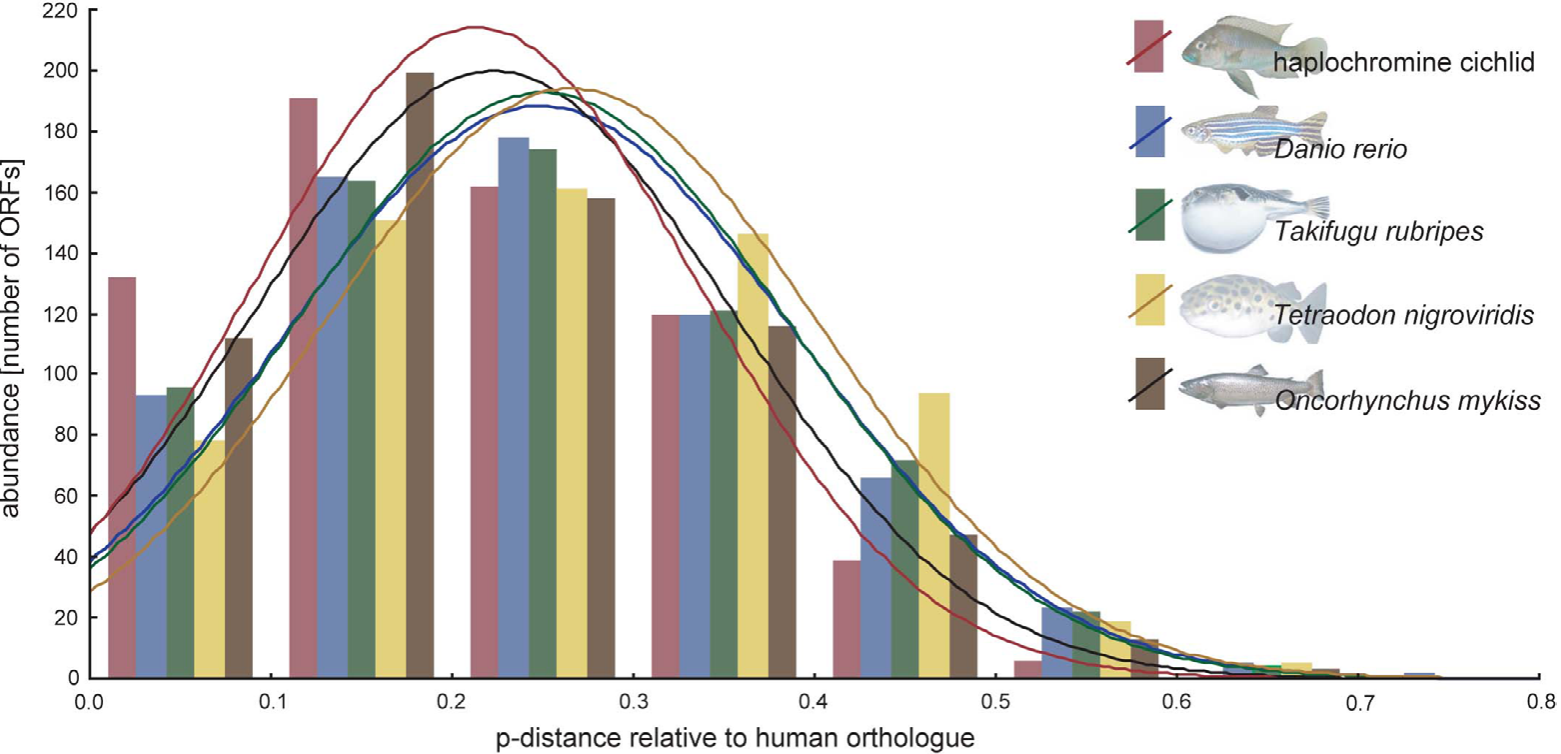
**Histogram of the abundance of amino acid sequence divergences of all five fish species (haplochromine cichlid, *Danio rerio, Takifugu rubripes, Tetraodon nigroviridis, and Oncorhynchus mykiss*) with respect to human genes**. P-distances have been calculated for a set of 759 ORFs found in all five fish species and plotted in categories of 0.1.

A histogram of the abundance of amino acid sequence divergences of all five fish species with respect to homologous human genes is depicted in Fig. 3. The p-distances appear normally distributed. With 0.211, cichlids show the lowest average distance followed by *Oncorhynchus mykiss *(0.216), *Danio rerio *(0.239), *Takifugu rubripes *(0.242), and *Tetraodon nigroviridis *(0.258). The average distance of all five fish species to *Homo sapiens *is 0.233. We also used the 482 redundant sequences that were found in all three large haplochromine cichlid EST datasets (*P. chilotes *and *P. sp. *"redtail sheller" [30]; *Astatotilapia burtoni*, this study) to calculate mean pairwise p-distances. Within these three cichlid species, we found a mean p-distance of 0.14 between *A. burtoni *and *P. chilotes*, 0.17 between *A. burtoni *and *P. sp. *"redtail sheller", and 0.08 between the two Lake Victoria species *P. chilotes *and *P. sp. *"redtail sheller".

We then calculated K_a_/K_s _ratios for all genes with a higher or slower rate of base substitution in cichlids. K_a_/K_s _ratios greater than one, which are indicative of positive selection in that gene, were found in four genes that evolve more slowly in cichlids compared to the other fish species. The highest K_a_/K_s _ratio (3.77) was found in the neuroendocrine *convertase subtilisin/kexin type 1 *that is responsible for processing large precursor proteins into mature peptide hormones [55,56]. In *claudin 3*, a member of the claudin family involved in the formation of tight junctions in various tissues [57], the K_a_/K_s _ratio was 1.55. A K_a_/K_s _ratio of 1.30 was observed in the catalyzing enzyme *glutathione peroxidase 3*, and a ratio of 1.19 was found in *ménage a trois 1 *(MNAT1), which is a member of the CDK7-cyclin H complex that functions in cell cycle progression [58], basal transcription, and DNA repair.

## Discussion

Expressed sequence tags are important genomic resources and their numbers in public databases such as GenBank are rapidly increasing. Full-length cDNA and EST sequencing projects typically accompany genome sequencing projects, as these data are essential for the recognition and annotation of genes, the characterization of the transcriptome, the identification of intron-exon boundaries and the detection of splice variants in eukaryotes, etc.[33,34,59-61]. In addition, the standardized procedure of cDNA library construction and normalization, and the comparably low costs of large-scale DNA sequencing facilitate EST projects in organisms for which the whole genome sequencing has not (yet) been completed. Thus, EST sequencing projects outnumber genome-sequencing projects – particularly in groups with larger genome sizes such as plants and vertebrates – leading to a large body of sequence data available for comparative analyses. Large-scale EST analyses have been used in many other contexts, such as primary gene expression assays [62,63], the estimation of the total number of genes in an organism [64], cDNA microarray annotations [65], or the construction of genetic linkage maps [66-68]. Expressed sequence tags can further be used for phylogenomics [36,69], and for the identification of microRNAs [70].

Despite their many advantages, there are also some problems associated with ESTs. For example, EST sequences typically cover only parts of a gene, so that two sequences of the same gene might not necessarily overlap. That only fragments of a gene are available also leads to problems with homology-based analyses such as BLAST. Then, EST sequences often contain the untranslated regions (UTRs) that are present in mRNAs but do not translate into amino acids. Finally, it is often difficult to figure out the proper reading frame, particularly in shorter ESTs, which impedes certain analyses. A combination of multiple EST projects (as we have done here) helps to alleviate some of the shortcomings inherent in EST data.

We have sequenced, annotated and conducted evolutionary analysis of ESTs of haplochromine cichlids for several reasons. First, this large set of sequence data for cichlid ORFs provides insight into the genome of a representative of haplochromine cichlids, which are a main model system for the study of adaptive evolution and explosive speciation [1-3]. Second, we wanted to extend the existing genomic resources for *Astatotilapia burtoni *such as a genomic BAC library [27] by establishing cDNA libraries from different tissues. Furthermore, these cDNA libraries provide the basis for annotated cDNA microarrays that are being used for expression analyses in a variety of cichlid species [22,28,71]. Finally, we were interested in identifying genes with a different evolutionary rate in the rapidly radiating cichlid lineage compared to other fish species, as well as in identifying genes that show the signature of adaptive evolution in cichlids.

Of the two *A. burtoni *cDNA libraries that were used for EST sequencing, the normalized mixed tissue library ('*pinky*') was of better quality. Not only were there much fewer redundant sequences as compared to the *brain *library, which was mainly due to the normalization step, but also the average insert size was larger and the average read length was longer. Altogether, about 85% of the sequenced cDNA clones led to high-quality ESTs of a length of >200 bp (86% in *pinky*, and 85% in *brain*). In the BLAST searches against *Takifugu rubripes*, *Tetraodon nigroviridis*, and *Danio rerio*, between 14% (when compared to *T. rubripes*; e-value ≤ 10^-50^) and 43% (when compared to *D. rerio*; e-value ≤ 10^-5^) of the *A. burtoni *ESTs led to hits (Fig. 1). This lies well within the range of other EST sequencing projects [63,65,72].

About 8,600 *A. burtoni *ORFs (or 75% of the high quality ESTs) were longer than 400 bp, and about 3,000 sequences could unambiguously be annotated and classified following the vocabulary provided by the Gene Ontology Consortium [Additional files 1, 2, 3, 4, 5, 6]. According to the Gene Ontology classification, it appears that a broad range of genes involved in functions, processes and compartments are represented in our EST set. This cichlid specific GO slim offers several advantages. First, it offers a rapid visual interpretation of gene subsets. Second, because the cichlid specific slim is built from those sequences used to build a cDNA microarray, it offers maximal power when testing for over- or under-representation of gene lists while reducing the need for correction for multiple hypothesis testing. Finally, it allows for a less experimenter-biased interpretation of microarray results, or other genomics analyses in a manner that can be easily compared between experiments.

One of our main goals was to characterize genes in haplochromine cichlids that show a faster or slower rate of base substitutions in cichlids compared to other fish species, as this is indicative of a relaxed or reinforced selection regime, respectively [35]. To this end, we combined our newly generated ESTs with previously published sequences for Lake Victoria haplochromine cichlids [30] and about 1,000 sequences obtained from a *Metriaclima zebra *skin cDNA library, which resulted in a total of about 45,000 ORFs. By means of homology searches against human, the two pufferfishes, trout, and zebrafish using local BLAST, we identified a set of 759 ORFs that are present in all species and that show a sufficient degree of homology (e-value ≤ 10^-50^) for further analyses with EverEST [37]. The number of genes with a cichlid-specific faster or slower rate of molecular evolution (always with human as outgroup) varied when different fish taxa were used in addition to the cichlid ORFs. However, we found a set of 170 genes (48 "faster" and 122 "slower"; Additional files 7, 8) that appeared in all comparisons and are, thus, good candidates for playing an important role in the evolution of (haplochromine) cichlid fishes.

When characterizing these genes further, by means of calculating K_a_/K_s _ratios, we found that four genes (or 2.35% of all deviating genes) showed the signature of adaptive evolution in the haplochromine lineage. The highest K_a_/K_s _ratio (3.77) was found in the neuroendocrine *convertase subtilisin/kexin type 1*, followed by *claudin 3*, (1.55), *glutathione peroxidase 3 *(1.50), and *ménage a trois 1 *(1.19). All gene fragments that show a K_a_/K_s _> 1 are found among the more slowly evolving genes. These genes are now candidate genes for further investigations. The gene with the highest K_a_/K_s _ratio appears particularly interesting. It is known that neuroendocrine factors, such as gonadotropin releasing hormone (GnRH), are involved in regulation of reproduction and behavior in *A. burtoni *[56,73].

In order to generate hypotheses regarding possible mechanisms by which the rapidly or slowly evolving cichlid genes might contribute to the process of adaptive radiation, we made use of the GO term annotations and cichlid specific slim. Over- and under-represented terms were identified among the annotations for the rapidly and slowly evolving cichlid genes (Table 2). Among the 759 ORFs for which p-distances were calculated, over 6,000 total annotations were applied to 647, 675, and 619 ORFs according to biological process, molecular function, and cellular component respectively. Therefore the majority of the 122 slowly evolving and 48 rapidly evolving genes could be classified bioinformatically.

**Table 2:** Gene Ontology terms which are over- or under-represented among the rapidly or slowly evolving cichlid ORFs. Hypergeometic p-values are reported uncorrected for multiple testing. The number of ORFs of deviating evolutionary rate (#) relative to the number of core set ORFs (total) is given.

**Representation**	**GO-ID**	**p-value**	**#**	**total**	**Description**
	**biological process**		**42 with higher p-distance (647 annotated)**		
over	none				
under	GO:0050896	0.0161	1	86	response to stimulus
	GO:0009987	0.0439	12	273	cellular process
	**molecular function**		**44 with higher p-distance (675 annotated)**		
	none				
	**Cellular component**		**40 with higher p-distance (619 annotated)**		
over	GO:0015629	0.0327	6	39	actin cytoskeleton
under	none				
	**biological process**		**103 with lower p-distance (647 annotated)**		
over	GO:0009987	0.0024	57	273	cellular process
over	GO:0007243	0.0052	8	19	protein kinase cascade
over	GO:0007155	0.0205	7	19	cell adhesion
over	GO:0040007	0.0208	6	15	growth
over	GO:0007154	0.0230	25	109	cell communication
over	GO:0007267	0.0071	7	16	cell-cell signaling
over	GO:0016477	0.0290	5	12	cell migration
over	GO:0040008	0.0290	5	12	regulation of growth
over	GO:0007409	0.0308	3	5	axonogenesis
over	GO:0007610	0.0308	3	5	behavior
over	GO:0015674	0.0308	3	5	di-, tri-valent inorganic cation transport
over	GO:0019752	0.0376	10	35	carboxylic acid metabolic process
over	GO:0007067	0.0402	4	9	mitosis
over	GO:0007417	0.0402	4	9	central nervous system development
under	GO:0008152	0.0016	63	477	metabolic process
under	GO:0046907	0.0180	2	44	intracellular transport
under	GO:0045045	0.0295	0	20	secretory pathway
under	GO:0009117	0.0421	0	18	nucleotide metabolic process
	**molecular function**		**110 with lower p-distance (675 annotated)**		
over	GO:0004930	0.0157	4	7	G-protein
over	GO:0003774	0.0233	6	15	motor activity
over	GO:0005262	0.0264	2	2	calcium channel
over	GO:0008047	0.0324	6	16	enzyme activator activity
over	GO:0005509	0.0333	12	43	calcium ion binding
over	GO:0019899	0.0435	4	9	enzyme binding
under	GO:0005525	0.0116	1	36	GTP binding
under	GO:0005198	0.0407	8	85	structural molecule activity
under	GO:0051082	0.0467	0	17	unfolded protein binding
under	GO:0003743	0.0467	0	17	translation initiation factor activity
under	GO:0003924	0.0481	1	27	GTPase activity
under	GO:0003676	0.0483	17	147	nucleic acid binding
	**cellular component**		**97 with lower p-distance (619 annotated)**		
over	GO:0016021	0.0096	22	88	integral to membrane
over	GO:0015630	0.0388	5	13	microtubule cytoskeleton
over	GO:0005625	0.0479	6	18	soluble fraction
over	GO:0005615	0.0479	6	18	extracellular space
under	GO:0032991	0.0001	19	222	macromolecular complex
under	GO:0043234	0.0015	18	195	protein complex
under	GO:0043226	0.0089	56	425	organelle
under	GO:0030529	0.0139	4	65	ribonucleoprotein complex
under	GO:0005829	0.0267	3	51	cytosol
under	GO:0005739	0.0311	6	75	mitochondrion

There was a relatively even distribution of rapidly evolving genes across all GO categories. Only three terms, "response to stimulus", "cellular process" and "actin cytoskeleton" deviated significantly from the distribution expected by chance alone. The most significant disproportionate under-representation for the rapidly evolving genes was the category of response to stimulus for which only 1 of the 86 possible annotated ORFs was included on the list.

The distribution across GO categories was highly non-uniform for the slowly evolving genes. Many categories from each ontology were represented by significantly more or fewer ORFs than would be expected by chance. Among those terms over-represented we found several relating to cellular processes such as protein kinase cascade, mitosis, and cell signaling as well as growth and cell adhesion, while metabolic process was under-represented along with the secretory pathway category.

The GO analysis highlights the possible categories of genes that may play an important role in the evolution of the haplochromine cichlid fishes. This analysis presents hypotheses to be tested through focused experimental or sequence analysis. An interesting contrast in GO analysis results was observed between the rapidly evolving genes that showed little tendency to derive from a particular class and slowly evolving genes that were more structured in their distribution. The lack of structure to the distribution of rapidly evolving genes may reflect the possibility that specialization among cichlids occurs along diverse biological pathways rather than a repeated divergence of a given biological process or molecular function. The GO categories that are over-represented among slowly evolving genes could represent genes whose functions are important for phenotypic plasticity or other traits linked to the successful adaptive radiation, while those categories that are under-represented by slowly evolving genes represent categories that are not as tightly constrained.

Our p-distance comparisons between the five fish species and human (as outgroup) also revealed that cichlids show the lowest average p-distance compared to *Homo sapiens *(Fig. 3). This might be an artifact that is due to the use of the haplochromine cichlid sequence as query for all BLAST searches. Alternatively, as we also found 122 slowly evolving genes in haplochromine cichlids, there might be a tendency in haplochromines to retain ancestral forms and functions. The pairwise average p-distance comparisons between the three cichlid species *Paralabidochromis chilotes, Ptyochromis sp. *"redtail sheller", and *Astatotilapia burtoni *revealed that the coalescence time between the two Lake Victoria species (0.08) is about half compared to their coalescence time with *A. burtoni *(0.14 and 0.17, respectively), which is in concordance to the phylogenetic relationships between these three taxa [4].

## Conclusion

Here we report the sequencing and annotation of more than 11,000 ESTs from the East African haplochromine cichlid *Astatotilapia burtoni*. Our EST set comprises a broad range of genes involved in functions, processes and compartments. By combining the *A. burtoni *ESTs with publicly available ORFs from two Lake Victoria haplochromines and subsequent comparisons to other fish model systems, we identify a set of 170 genes with haplochromine-specific differences in evolutionary rates. These genes appear as good candidates for playing an important role in the evolution of the exceptional diversity found in (haplochromine) cichlids. Interestingly, genes that were more slowly evolving in the cichlid lineage were not evenly distributed across Gene Ontology categories; classes that are over-represented could represent genes whose functions are important for successful adaptive radiation. We also identify four genes with a K_a_/K_s _ratio greater than one, which are, hence, likely to have undergone positive selection in haplochromines. The *A. burtoni *ESTs provide novel insights into the genome of haplochromine cichlids and will serve as valuable resource for researchers working in the field of (cichlid) evolutionary genomics, particularly in the light of the forthcoming sequencing of four cichlid genomes.

## Methods

### Fishes

*Astatotilapia burtoni *were kept at Stanford, and at the Tierforschungsanlage of the University of Konstanz under standard conditions (12 h light, 12 h dark; 26°C). For RNA isolation, fishes were sacrificed after anesthetization with MS 222 (Sigma).

### Pinky cDNA Library Construction

For the preparation of the pinky cDNA library, total RNA was isolated from the following tissues of adult *A. burtoni*: brain, caudal fin, anal fin (male), lips, muscle, ovary (female), and skin. Additionally, we isolated total RNA from a juvenile individual (about 30 days after fertilization). Total RNA was isolated by guanidine thiocyanate/phenol-chlorophorm-isoamyl alcohol extraction and lithium-chloride precipitation. The different RNA samples were pooled and cDNA was synthesized using the SMART PCR cDNA Synthesis Kit (Clontech) following the manufacturer's protocol. Amplified cDNA was purified using the QIAquick PCR Purification Kit (Qiagen) and concentrated by ethanol precipitation. The pellet was dissolved in 10 μl H_2_O. For normalization, three microliters of purified cDNA were mixed with 1 μl hybridization buffer (200 mM HEPES-HCl, pH 8.0; 2 M NaCl) and incubated at 95°C for 5 minutes and at 70°C overnight. Then, 1 μl of DNAse buffer (500 mM Tris-HCl, pH 8.0; 50 mM MgCl_2_, 10 mM DTT) and 0.5 μl of DSN enzyme (duplex-specific nuclease; Evrogen, Russia) were added, and the mix was incubated at 65°C for 20 minutes. The normalization reaction was terminated by adding 1 μl 50 mM EDTA and incubation at 95°C for 7 minutes. Normalized cDNA was PCR amplified (20 cycles) and cloned into pAL 16 vectors.

### Brain cDNA Library Construction

A full-length, directional (EcoRI – XhoI) cDNA library was constructed in Lambda ZapII phage vector (Stratagene) with mRNA from *A. burtoni *brains (both sexes at all stages of development and social condition were included). Construction of this library has previously been described in [22]. For cDNA sequencing, we used 2 μl of purified PCR products, which were also used for the construction of a cDNA microarray [22].

### DNA-sequencing and Sequence Analysis

For sequencing of the normalized pinky cDNA library we used purified plasmid DNA from 1 ml colonies that were grown overnight. Plasmid DNA was directly sequenced using T7 primers and the BigDye Termination Reaction Kit v3.0 (Applied Biosystems) on ABI 3730 and ABI 3100 automated capillary DNA sequencers (Applied Biosystems). Sequences of the brain cDNA library were determined on an ABI 3100 DNA sequencer after cycle sequencing reactions from purified PCR products that were available from the construction of a cDNA microarray [22] using the primer CSVP3 (5'-AAGCGCGCAATTAACCCTCACTA-3') and the BigDye Termination Reaction Kit v3.0 (Applied Biosystems).

Base-calling and quality trimming were performed with phred [74] using a quality score > 20. Vectors were trimmed with Sequencher 4.2.2 (Genecodes). Those ESTs having a total length of >200 bp after quality and vector trimming were considered "high-quality ESTs". Screens for possible contaminations were conducted by blastn searches against the *E. coli *genome, and the EST_human, EST_mouse and EST_others databases (downloaded in March 2005). Sequences have been deposited in GenBank under accession numbers CN468542 – CN472211 (brain library) and DY625779 – DY632420 (pinky library).

### Annotation of *A. burtoni *ESTs

High quality *A. burtoni *ESTs were screened by tblastx searches against protein data from *Danio rerio *(Zebrafish Sequencing Group at the Sanger Institute), *Homo sapiens *(GenBank) and *Takifugu rubripes *(JGI Fugu v3.0) as well as ESTs from *Oncorhynchus mykiss *and *Tetraodon nigroviridis *(GenBank) using the standard vertebrate code for translation into amino acids. The expected value thresholds (e-values) were set to < 1 × 10^-5^, < 1 × 10^-15^, and < 1 × 10^-50^. The proper open reading frame for *A. burtoni *ESTs was determined with EverEST [37], based on the results from these BLAST searches.

For functional annotation of *A. burtoni *ESTs, we followed the vocabulary provided by the Gene Ontology Consortium using the GO database [75]. Gene Ontology terms were applied to the cichlid assembled sequences by BLAST comparison to the Gene Ontology database (release 200704), which represents protein sequence for all contributed genes for which at least one GO annotation has been applied based on experimental evidence rather than only inferred electronic annotation of sequence. All GO annotations at any confidence level were then transferred from the single best-hit gene using e-value < 10^-12 ^as a threshold. The collection of GO terms used was "slimmed" in order to produce useful summaries of the annotations.

This cichlid specific slim [Additional files 4, 5, 6] is based upon statistical consideration for analysis of microarray results. The leaf most nodes have been selected for which 20 or more *A. burtoni *assembled sequences were annotated with this term. Parent nodes were retained only when an additional 20 *A. burtoni *assembled sequences were included. To assess the enrichment of particular classes of genes among the genes showing deviating rate of molecular evolution, Gene Ontology annotation terms were tested for significant over- and under-representation in either the higher or lower p-distance list using a hypergeometric test implemented in the BINGO plugin [76] for Cytoscape [77]. Due to the exploratory nature of this analysis and controversial application of correction techniques [78], reported p-values are not corrected for multiple testing. Only the representation for the leaf most node is reported except in cases when a larger, parent node showed increased significance. The directed acyclic graphs (DAGs) were created using hierarchical visualization in Cytoscape and manually adjusted to facilitate comprehension.

### Evolutionary Analyses

For evolutionary analyses of ESTs from haplochromine cichlids, we combined our newly generated high-quality ESTs from *A. burtoni *with previously published ESTs from *Paralabidochromis chilotes *and *Ptyochromis sp. *"redtail sheller" [30] and with about 1,000 ESTs obtained from a cDNA library made from *Metriaclima zebra *skin tissue (W. Salzburger, H. A. Hofmann & A. Meyer, unpublished). The combined dataset, including more than 45,000 ESTs, was BLASTed against protein data from *Danio rerio*, *Homo sapiens *and *Takifugu rubripes *as well as ESTs from *Oncorhynchus mykiss *and *Tetraodon nigroviridis *(see above for source of data) using the translated BLAST routine and the standard vertebrate code. This was done to identify a set of ORFs present in all datasets under study. BLAST searches were performed with an e-value of < 1 × 10^-50 ^in order to achieve high levels of confidence in the similarity searches. The cichlid query sequences and the best hits from every single BLAST search against the different databases were imported into EverEST [37].

In order to identify coding sequences showing a deviating rate of molecular evolution in haplochromine cichlids compared to other fish lineages we applied the triangle method implemented in EverEST. In this approach, the query sequences are aligned to their best BLAST hits in two ingroup and one outgroup taxa using the T-Coffee algorithm [79] as implemented in EverEST [37]. This reveals multiple sequence alignments consisting of four taxa. Then, uncorrected pairwise p-distances are calculated for all taxon pairs in each alignment, which are used to construct neighbor-joining trees and, after rooting with the outgroup sequences, for a global ternary representation. A relative rate test was applied to each of the orthologous groups. We applied the nonparametric rate test developed by Tajima [80], and compared the genes with their human and their fish orthologs in order to identify higher or lower substitution rates.

For these analyses, we used the human sequences as outgroup since tetrapods are valid outgroup taxa for teleost fish and the human genome is the most complete and best annotated genome among those. In addition to our haplochromine cichlid query sequences, we used different sets of ingroup taxa in order to minimize biasing effects due to sparse taxon sampling. We used the following combinations of taxa for our evolutionary rate analyses using 759 ORFs that have been found in all datasets: (human, (haplochromine cichlid, *Danio rerio*, *Takifugu rubripes*)) (Fig. 2a), (human, (haplochromine cichlid, *Danio rerio*, *Tetraodon nigroviridis*)) (not shown), (human, (haplochromine cichlid, *Danio rerio*, *Oncorhynchus mykiss*)) (not shown). As a control, we also analyzed a data set without the cichlid-query sequences for the same set of ORFs (human, (*Danio rerio*, *Oncorhynchus mykiss*, *Takifugu rubripes*)) (Fig. 2b). We note that this approach might lead to an underestimation of the number of faster evolving genes, as genes that accumulated too many mutations are likely not to be chosen in the stringent initial BLAST searches. We would also like to point out that some of the observed rate differences might have accumulated on the evolutionary lineage leading to the cichlids but before the cichlids have evolved as a group.

For orthologous groups, where the p-distance in the haplochromine cichlids were significantly (p < 0.05) higher or lower compared to other fish, the ratio of the number of nonsynonymous substitutions per nonsynonymous site (K_a_) to the number of synonymous substitutions per synonymous site (K_s_) was calculated based on a likelihood approach [81] to evaluate the selective forces acting on those proteins. The K_a_/K_s _ratio is an indicator of the form of sequence evolution, with K_a_/K_s _>> 1 providing strong evidence that positive selection has acted to change the protein sequence.

We also constructed a histogram of amino acid sequence divergence of all five fish datasets with respect to homologous human sequences. We finally used the redundant sequences in the three datasets *P. chilotes*, *P. sp. *"redtail sheller", and *A. burtoni *to calculate pairwise average p-distances.

## Abbreviations

DAG, directed acyclic graph; EST, expressed sequence tag; GO, gene ontology; ORF, open reading frame

## Authors' contributions

WS, HAH and AM designed the study. WS and HAH were involved in library construction; WS and IB carried out the molecular work; WS, DS, SCPR, and IB performed the analyses. All authors contributed to the preparation of the manuscript. They read and approved the final version.

## Supplementary Material

Additional file 1 **Gene ontology table (generic GO slim subset for molecular function)**. Hierarchical classification of the GO slim subset for molecular function. Indented terms are children of parent terms listed above. For each term, the number of *A. burtoni *assembled sequences that match genes to which Gene Ontology annotations have been assigned at, or below, this general level is given. Note that genes may be assigned to more than one term and child terms may have more than one parent term. For parent terms, the total number of *A. burtoni *assembled sequences is given in parentheses. Match means that the annotation derives from a gene that was the "best hit" for the *A. burtoni *sequence at and e-value < 10^-12^. Click here for file

Additional file 2 **Gene ontology table (generic GO slim subset for biological process)**. Hierarchical classification of the GO slim subset for biological process. Indented terms are children of parent terms listed above. Genes may be assigned to more than one term. For each term, the number of *A. burtoni *assembled sequences that match genes to which Gene Ontology annotations have been assigned at, or below, this general level is given. Note that genes may be assigned to more than one term and child terms may have more than one parent term. For parent terms, the total number of *A. burtoni *assembled sequences is given in parentheses. Match means that the annotation derives from a gene that was the "best hit" for the *A. burtoni *sequence at and e-value < 10^-12^. Click here for file

Additional file 3 **Gene ontology table (generic GO slim subset for cellular component)**. Hierarchical classification of the GO slim subset for cellular component. Indented terms are children of parent terms listed above. Genes may be assigned to more than one term. For each term, the number of *A. burtoni *assembled sequences that match genes to which Gene Ontology annotations have been assigned at, or below, this general level is given. Note that genes may be assigned to more than one term and child terms may have more than one parent term. For parent terms, the total number of *A. burtoni *assembled sequences is given in parentheses. Match means that the annotation derives from a gene that was the "best hit" for the *A. burtoni *sequence at and e-value < 10^-12^. Click here for file

Additional file 4 **Directed acyclic graph (DAG) of the cichlid specific Gene ontology (GO) slim for molecular function**. The graph shows the cichlid specific GO slim for molecular function. Molecular function terms were selected for inclusion in the ontologies such that leaf nodes include approximately 20 annotated genes. Circle size represents relative number of genes annotated to each parent node. Click here for file

Additional file 5 **Directed acyclic graph (DAG) of the cichlid specific Gene ontology (GO) slim for biological process**. The graph shows the cichlid specific GO slim for biological process. Biological process terms were selected for inclusion in the ontologies such that leaf nodes include approximately 20 annotated genes. Circle size represents relative number of genes annotated to each parent node. Click here for file

Additional file 6 **Directed acyclic graph (DAG) of the cichlid specific Gene ontology (GO) slim for cellular component**. The graph shows the cichlid specific GO slim for cellular component. Cellular component terms were selected for inclusion in the ontologies such that leaf nodes include approximately 20 annotated genes. Circle size represents relative number of genes annotated to each parent node. Click here for file

Additional file 7 **ESTs with higher p-distances**. The table shows ESTs where the p-distance between *Homo sapiens *and haplochromine cichlid amino acid sequences is significantly higher as compared to other fish species (*Danio rerio, Takifugu rubripes, Tetraodon nigroviridis *and *Oncorhynchus mykiss*). Annotation means that the *Homo sapiens *gene was "best hit" for the cichlid sequence (and e-value < 10^-50^). Click here for file

Additional file 8 **ESTs with smaller p-distances**. The table shows ESTs where the p-distance between *Homo sapiens *and haplochromine cichlid amino acid sequences is significantly smaller as compared to other fish species (*Danio rerio*, *Takifugu rubripes*, *Tetraodon nigroviridis*, and *Oncorhynchus mykiss*). Annotation means that the *Homo sapiens *gene was "best hit" for the Cichlid sequence (and e-value < 10^-50^). Click here for file
