# Supplementary material for: Annotation of expressed sequence tags for the East African cichlid fish Astatotilapia burtoni and evolutionary analyses of cichlid ORFs
Source: BMC Genomics. 2008 Feb 25;9:96. doi: 10.1186/1471-2164-9-96 (PMC2279125; doi:10.1186/1471-2164-9-96)
Supplement: Additional file 1 — Gene ontology table (generic GO slim subset for molecular function). Hierarchical classification of the GO slim subset for molecular function. Indented terms are children of parent terms listed above. For each term, the number of A. burtoni assembled sequences that match genes to which Gene Ontology annotations have been assigned at, or below, this general level is given. Note that genes may be assigned to more than one term and child terms may have more than one parent term. For parent terms, the total number of A. burtoni assembled sequences is given in parentheses. Match means that the annotation derives from a gene that was the "best hit" for the A. burtoni sequence at and e-value < 10-12. [file 1471-2164-9-96-S1.PDF]

Molecular Function	2692	
antioxidant activity	18	
binding	684	(2097)
calcium ion	154	
carbohydrate binding	43	
chromatin binding	21	
lead ion binding	0	
lipid binding	82	
nucleic acid binding	130	(586)
DNA binding	193	(227)
transcription factor activity	89	
RNA binding	293	
translation factor activity, nucleic acid binding	55	
nucleotide binding	459	
oxygen binding	24	
protein binding	1096	(1183)
cytoskeletal protein binding	52	(110)
actin binding	65	
receptor binding	110	
triplet codon-amino acid adaptor activity	0	
catalytic activity	574	(1239)
electron carrier activity	98	
hydrolase activity	396	(473)
nuclease	9	
peptidase activity	117	
phosphoprotein phosphatase	25	
transferase activity	229	(277)
kinase activity	103	(125)
protein kinase activity	55	
chaperone regulator activity	4	
enzyme regulator activity	127	
motor activity	35	
nutrient reservoir activity	0	
protein tag 1		
signal transducer activity	63	(170)
receptor activity	116	
structural molecule activity	382	
transcription regulator activity	116	(167)
translation regulator activity	5	(59)
transporter activity	338	(415)
ion channel activity	84	
neurotransmitter transporter activity	4	
