# Supplementary material for: Annotation of expressed sequence tags for the East African cichlid fish Astatotilapia burtoni and evolutionary analyses of cichlid ORFs
Source: BMC Genomics. 2008 Feb 25;9:96. doi: 10.1186/1471-2164-9-96 (PMC2279125; doi:10.1186/1471-2164-9-96)
Supplement: Additional file 2 — Gene ontology table (generic GO slim subset for biological process). Hierarchical classification of the GO slim subset for biological process. Indented terms are children of parent terms listed above. Genes may be assigned to more than one term. For each term, the number of A. burtoni assembled sequences that match genes to which Gene Ontology annotations have been assigned at, or below, this general level is given. Note that genes may be assigned to more than one term and child terms may have more than one parent term. For parent terms, the total number of A. burtoni assembled sequences is given in parentheses. Match means that the annotation derives from a gene that was the "best hit" for the A. burtoni sequence at and e-value < 10-12. [file 1471-2164-9-96-S2.PDF]

| **Biological Process** | **2532** | |
|---|---|---|
| anatomical structure morphogenesis | 131 | (152) |
| cell growth | 26 | |
| behavior | 24 | |
| cell communication | 9 | (437) |
| cell-cell signaling | 83 | |
| signal transduction | 381 | |
| cell cycle | 163 | |
| cell differentiation | 166 | (283) |
| cell death | 138 | |
| cell homeostasis | 132 | |
| cell proliferation | 88 | |
| cell recognition | 8 | |
| cellular component organization and biogenesis | 359 | (659) |
| cytoplasm organization and biogenesis | 1 | |
| organelle organization and biogenesis | 189 | (353) |
| cytoskeleton organization and biogenesis | 159 | |
| mitochondrion organization and biogenesis | 13 | |
| death | 0 | (138) |
| growth | 18 | (42) |
| metabolic process | 213 | (1731) |
| biosynthetic process | | (583) |
| translation | 313 | |
| catabolic process | 194 | |
| generation of precursor metabolites and energy | 82 | (299) |
| electron transport | 226 | |
| primary metabolic process | 0 | (1425) |
| amino acid and derivative metabolic process | 65 | |
| carbohydrate metabolic process | 123 | |
| lipid metabolic process | 81 | |
| nucleobase, nucleoside, nucleotide and nucleic acid metabolic process | 230 | (563) |
| DNA metabolic process | 146 | |
| transcription | 288 | |
| protein metabolic process | 335 | (805) |
| protein modification process | 240 | |
| secondary metabolic process | 83 | |
| multicellular organismal development | 330 | (365) |
| embryonic development | 67 | |
| regulation of biological process | 660 | |
| regulation of gene expression, epigenetic | 7 | |
| reproduction | 66 | |
| response to abiotic stimulus | 46 | |
| response to biotic stimulus | 34 | |
| response to endogenous stimulus | 68 | |
| response to external stimulus | 41 | |
| response to stress | 180 | |
| symbiosis, encompassing mutualism through parasitism | 1 | |
| transport | 522 | (746) |
| ion transport | 305 | |
| protein transport | 155 | |
| viral reproduction | 5 | |
