# Supplementary material for: Annotation of expressed sequence tags for the East African cichlid fish Astatotilapia burtoni and evolutionary analyses of cichlid ORFs
Source: BMC Genomics. 2008 Feb 25;9:96. doi: 10.1186/1471-2164-9-96 (PMC2279125; doi:10.1186/1471-2164-9-96)
Supplement: Additional file 3 — Gene ontology table (generic GO slim subset for cellular component). Hierarchical classification of the GO slim subset for cellular component. Indented terms are children of parent terms listed above. Genes may be assigned to more than one term. For each term, the number of A. burtoni assembled sequences that match genes to which Gene Ontology annotations have been assigned at, or below, this general level is given. Note that genes may be assigned to more than one term and child terms may have more than one parent term. For parent terms, the total number of A. burtoni assembled sequences is given in parentheses. Match means that the annotation derives from a gene that was the "best hit" for the A. burtoni sequence at and e-value < 10-12. [file 1471-2164-9-96-S3.PDF]

Cellular Component	2293	
cell	804	(2216)
intracellular	540	(1883)
chromosome	49	(62)
nuclear chromosome	20	
cilium	7	
cytoplasm	488	(1367)
cytoplasmic membrane-bound vesicle	61	
cytosol	343	
endoplasmic reticulum	104	
endosome	10	
Golgi	65	
microtubule organizing center	15	
mitochondrion	414	
peroxisome	8	
plastid	4	
ribosome	244	
vacuole	13	(39)
lysosome	27	
cytoskeleton	204	(216)
nucleus	576	(630)
nuclear envelope	31	
nucleolus	60	
nucleoplasm	87	
plasma membrane	252	
extracellular region	62	(184)
extracellular space	134	
proteinaceous extracellular matrix	35	
organelle	17	(1538)
protein complex	808	
unlocalized protein complex	1	
