# Supplementary figures and images for: Annotation of expressed sequence tags for the East African cichlid fish Astatotilapia burtoni and evolutionary analyses of cichlid ORFs
Source: BMC Genomics. 2008 Feb 25;9:96. doi: 10.1186/1471-2164-9-96 (PMC2279125; doi:10.1186/1471-2164-9-96)

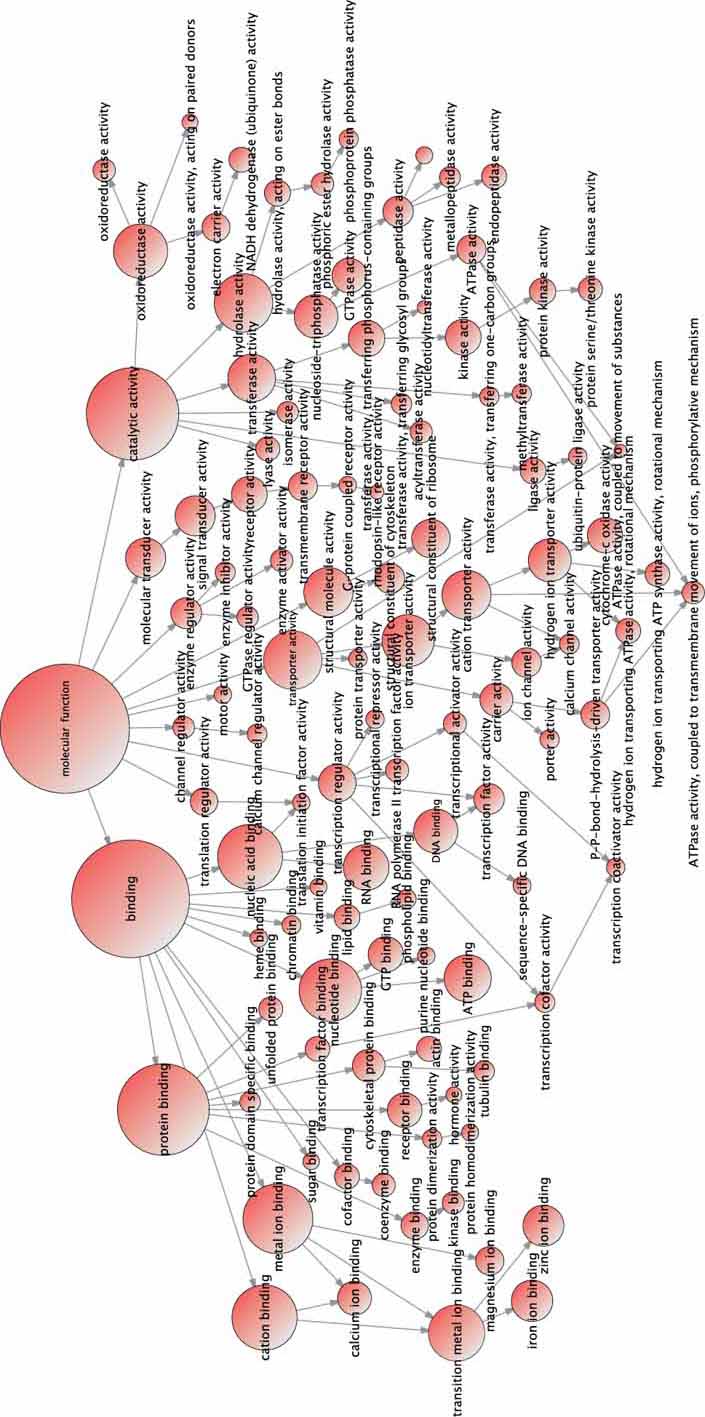

Supplement: Additional file 4 — Directed acyclic graph (DAG) of the cichlid specific Gene ontology (GO) slim for molecular function. The graph shows the cichlid specific GO slim for molecular function. Molecular function terms were selected for inclusion in the ontologies such that leaf nodes include approximately 20 annotated genes. Circle size represents relative number of genes annotated to each parent node. [file 1471-2164-9-96-S4.JPEG]

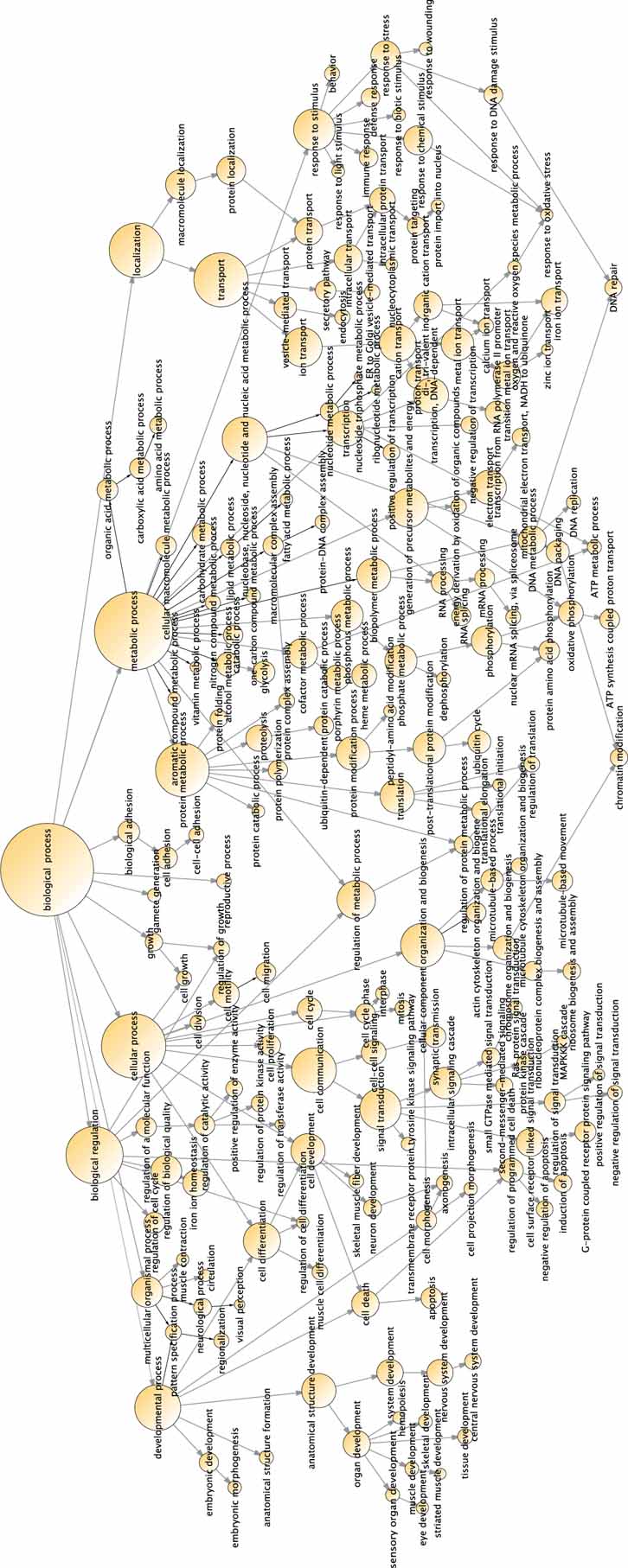

Supplement: Additional file 5 — Directed acyclic graph (DAG) of the cichlid specific Gene ontology (GO) slim for biological process. The graph shows the cichlid specific GO slim for biological process. Biological process terms were selected for inclusion in the ontologies such that leaf nodes include approximately 20 annotated genes. Circle size represents relative number of genes annotated to each parent node. [file 1471-2164-9-96-S5.JPEG]

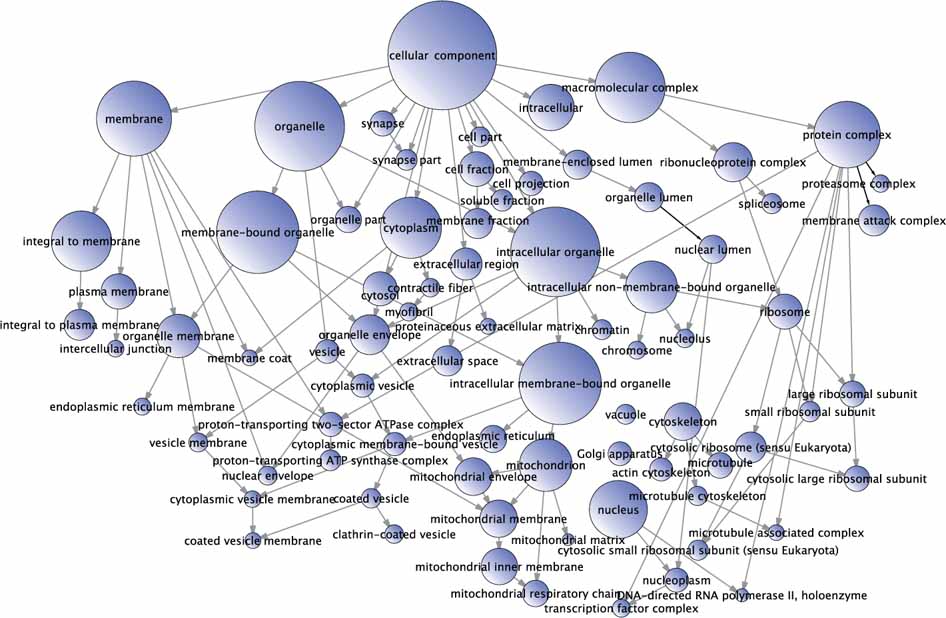

Supplement: Additional file 6 — Directed acyclic graph (DAG) of the cichlid specific Gene ontology (GO) slim for cellular component. The graph shows the cichlid specific GO slim for cellular component. Cellular component terms were selected for inclusion in the ontologies such that leaf nodes include approximately 20 annotated genes. Circle size represents relative number of genes annotated to each parent node. [file 1471-2164-9-96-S6.JPEG]
