# Supplementary material for: Annotation of expressed sequence tags for the East African cichlid fish Astatotilapia burtoni and evolutionary analyses of cichlid ORFs
Source: BMC Genomics. 2008 Feb 25;9:96. doi: 10.1186/1471-2164-9-96 (PMC2279125; doi:10.1186/1471-2164-9-96)
Supplement: Additional file 7 — ESTs with higher p-distances. The table shows ESTs where the p-distance between Homo sapiens and haplochromine cichlid amino acid sequences is significantly higher as compared to other fish species (Danio rerio, Takifugu rubripes, Tetraodon nigroviridis and Oncorhynchus mykiss). Annotation means that the Homo sapiens gene was "best hit" for the cichlid sequence (and e-value < 10-50). [file 1471-2164-9-96-S7.PDF]

Homo sapien best hit	GenBank acc. cichlid	Ka/Ks ratio	p-dist.
|NP_742034.1| retinol dehydrogenase 10	BJ671564	0.0035	0.27
|NP_057267.2| ribosomal protein P0-like protein; ribosomal protein, large,	CN470249	0.0038	0.26
|NP_000017.1| adenylosuccinate lyase; adenylosuccinase	BJ673035	0.0044	0.251
|XP_030559.1| PREDICTED: PAR-6 beta	BJ697326	0.0044	0.272
|NP_003272.2| troponin I, skeletal, slow; Troponin-I, skeletal, slow	DY629797	0.0044	0.385
|NP_001210.1| calumenin precursor	DY627455	0.0072	0.424
|NP_003583.2| cullin 1	CN468603	0.0219	0.317
|NP_059133.1| cleavage and polyadenylation specific factor 2; cleavage and	BJ683745	0.0267	0.25
|NP_009097.1| phosphatidylinositol-binding clathrin assembly protein; clathrin	BJ691629	0.0295	0.469
|NP_001959.1| eukaryotic translation initiation factor 4E; eukaryotic translation	BJ686392	0.0302	0.306
|NP_002063.2| guanine nucleotide binding protein (G protein), q polypeptide	BJ698454	0.0432	0.301
|NP_056170.1| joined to JAZF1	CN469599	0.0438	0.257
|NP_036265.1| coatomer protein complex, subunit gamma 2; coat protein,	BJ672808	0.0484	0.272
|NP_006764.3| DEAD (Asp-Glu-Ala-Asp) box polypeptide 18; Myc-regulated DEAD box	BJ700024	0.0547	0.286
|NP_055205.1| staphylococcal nuclease domain containing 1; EBNA-2 co-activator	BJ673580	0.0561	0.255
|NP_060104.2| potassium channel tetramerisation domain containing 9	BJ687766	0.0564	0.331
|NP_001737.1| calnexin	BJ685700	0.0574	0.4
|NP_006612.2| S-adenosylhomocysteine hydrolase-like 1; S-adenosyl homocysteine	DY632021	0.061	0.374
|NP_036205.1| chaperonin containing TCP1, subunit 5 (epsilon) [Homo sapiens]	BJ698817	0.0619	0.275
|NP_003679.1| calcium/calmodulin-dependent serine protein kinase (MAGUK family)	BJ682470	0.0639	0.286
|NP_612639.1| taube nuss; TAF8 RNA polymerase II, TATA box binding protein	BJ703081	0.0664	0.298
|NP_114366.1| poly(rC)-binding protein 2 isoform b; alpha-CP2; poly(rC)-binding	CN468947	0.0699	0.327
|NP_036475.2| nicotinamide nucleotide transhydrogenase	DY629769	0.0733	0.325
|NP_003926.1| topoisomerase (DNA) III beta; topoisomerase III beta	BJ701292	0.0736	0.328
|NP_060802.1| DDX19-like protein; RNA helicase	DY625929	0.0757	0.256
|NP_689953.1| 3-hydroxyisobutyrate dehydrogenase	BJ690821	0.0759	0.282
|NP_005854.2| neuroepithelial cell transforming gene 1; guanine nucleotide	BJ680834	0.0772	0.28
|NP_003793.1| myosin, heavy polypeptide 13, skeletal muscle; extraocular muscle	BJ679618	0.0778	0.318
|NP_003306.1| DnaJ (Hsp40) homolog, subfamily C, member 7; tetratricopeptide	BJ676351	0.0798	0.31
|NP_078939.3| aminopeptidase-like 1	DY629835	0.0836	0.254
|NP_002586.2| PCTAIRE protein kinase 2; serine/threonine-protein kinase	BJ703098	0.0868	0.286
|NP_001410.2| ELAV-like 1; embryonic lethal, abnormal vision, drosophila,	BJ685085	0.0871	0.25
|NP_003743.1| eukaryotic translation initiation factor 3, subunit 8, 110kDa;	DY626506	0.0895	0.434
|NP_002289.1| L-plastin; plastin 2; Lymphocyte cytosolic protein-1 (plasmin)	BJ696087	0.0906	0.283
|NP_002070.1| aspartate aminotransferase 1	BJ700931	0.0944	0.263
|NP_057388.1| ribosomal protein L24-like; homolog of yeast ribosomal like protein	DY625961	0.1002	0.323
|NP_000174.1| hydroxyacyl dehydrogenase, subunit B; 3-ketoacyl-Coenzyme A	DY627495	0.1026	0.269
|NP_002568.2| p21-activated kinase 2; S6/H4 kinase	BJ692414	0.1037	0.405
|NP_443133.1| solute carrier family 25 (mitochondrial carrier; phosphate	DY629151	0.1174	0.297
|NP_006383.2| nucleolar protein 5A; nucleolar protein 5A (56kD with KKE/D repeat)	DY626311	0.1251	0.25
|NP_001961.1| eukaryotic translation initiation factor 5A; eIF5AI	BJ692285	0.1317	0.273
|NP_004362.1| coatomer protein complex, subunit alpha; xenin; alpha coat protein	BJ690224	0.1395	0.286
|NP_115915.1| 5'-nucleotidase, cytosolic IA; cytosolic 5' nucleotidase, type 1A;	BJ701030	0.1501	0.379
|XP_377129.2| PREDICTED: similar to golgi autoantigen, golgin subfamily a, 7	BJ686655	0.1522	0.284
|NP_006400.2| actin related protein 2/3 complex subunit 1A; actin binding protein	DY630466	0.1682	0.359
|NP_006730.2| minichromosome maintenance deficient protein 5; DNA replication	BJ690838	0.1858	0.284
|NP_039234.1| chloride intracellular channel 4; chloride intracellular channel 4	BJ701465	0.1977	0.402
|NP_060103.1| chromosome 6 open reading frame 37; retinal expressed gene C6orf37	BJ674373	0.2952	0.259
