# Supplementary material for: Annotation of expressed sequence tags for the East African cichlid fish Astatotilapia burtoni and evolutionary analyses of cichlid ORFs
Source: BMC Genomics. 2008 Feb 25;9:96. doi: 10.1186/1471-2164-9-96 (PMC2279125; doi:10.1186/1471-2164-9-96)
Supplement: Additional file 8 — ESTs with smaller p-distances. The table shows ESTs where the p-distance between Homo sapiens and haplochromine cichlid amino acid sequences is significantly smaller as compared to other fish species (Danio rerio, Takifugu rubripes, Tetraodon nigroviridis, and Oncorhynchus mykiss). Annotation means that the Homo sapiens gene was "best hit" for the Cichlid sequence (and e-value < 10-50). [file 1471-2164-9-96-S8.PDF]

Homo sapien best hit	GenBank acc. cichlid	Ka/Ks ratio	p-distance
|NP_000276.1| Xaa-Pro dipeptidase; proline dipeptidase	DY628286	0	0.006
|NP_000060.1| calcium channel, voltage-dependent, L type, alpha 1S subunit; calcium	DY627704	0	0.025
|NP_002492.1| nuclear factor I/X (CCAAT-binding transcription factor)	BJ684881	0.0188	0.037
|NP_006574.1| peropsin	DY628165	0.6	0.04
|NP_694592.1| protein-tyrosine kinase fyn isoform b; proto-oncogene	CN469350	0.0121	0.044
|XP_371813.2| PREDICTED: kinesin family member C1	DY627797	0.5	0.051
|NP_742055.1| eyes absent 1 isoform b; Eyes absent, Drosophila, homolog of, 1;	BJ693416	0.0349	0.058
|NP_000977.1| ribosomal protein L24; 60S ribosomal protein L24; ribosomal protein	BJ684650	0.0419	0.059
|NP_001264.1| chromodomain helicase DNA binding protein 4; Mi-2b	BJ687855	0.0305	0.068
|NP_057399.1| GULP, engulfment adaptor PTB domain containing 1; engulfment	CN469384	0.0219	0.073
|NP_061133.1| peroxisomal membrane protein 2, 22kDa; peroxisomal membrane protein	DY628735	0.4231	0.074
|NP_004437.1| glutamyl-prolyl tRNA synthetase; glutamate tRNA ligase	BJ676918	0.012	0.08
|NP_001924.2| dihydrolipoamide S-succinyltransferase (E2 component of	BJ679359	0.0255	0.094
|NP_001369.1| dynein, cytoplasmic, intermediate polypeptide 2	BJ703324	0.0073	0.099
|NP_077022.1| mitogen-activated protein kinase associated protein 1; ras	DY626351	0.0353	0.101
|NP_073557.3| DiGeorge syndrome critical region gene 8	CN469327	0.0364	0.104
|NP_002075.2| plasma glutathione peroxidase 3 precursor	DY627877	1.3043	0.106
|NP_004495.2| HIV-1 Rev binding protein; nucleoporin-like protein RIP; Rab,	CN468756	0.0241	0.107
|NP_056067.1| NPF/calponin-like protein; EH domain-binding protein 1	CN469453	0.0015	0.108
|NP_003984.2| CDC-like kinase 2 isoform 1; dual specificity protein kinase CLK2;	BJ699169	0.0544	0.109
|NP_056371.1| signal-induced proliferation-associated 1 like 1; signal-induced	BJ668716	0.0063	0.11
|NP_000993.1| ribosomal protein P0; 60S acidic ribosomal protein P0; acidic	BJ701638	0.053	0.111
|NP_001297.1| claudin 3; Clostridium perfringens enterotoxin receptor 2; rat	DY631785	1.5556	0.115
|NP_004312.2| axonal transport of synaptic vesicles; kinesin, heavy chain, member	DY632057	0.0393	0.123
|NP_005494.2| amyloid beta A4 precursor protein-binding, family A, member 2;	CN469652	0.02	0.127
|NP_006304.1| ubiquitin specific protease 15; deubiquitinating enzyme	BJ693203	0.048	0.128
|NP_004969.2| Shaw-related voltage-gated potassium channel protein 4 isoform a;	DY631758	0.7808	0.13
|NP_057365.2| STE20-like kinase; STE2-like kinase	BJ685775	0.0266	0.137
|NP_783860.1| synaptotagmin IX	CN470765	0.0194	0.14
|NP_003281.1| tropomyosin 4	BJ674753	0.0384	0.145
|NP_000981.1| ribosomal protein L27a; 60S ribosomal protein L27a	BJ682982	0.0861	0.145
|NP_001261.1| chromodomain helicase DNA binding protein 1	BJ671483	0.0072	0.147
|NP_005075.2| phospholipase A2, group VII; platelet-activating factor	DY628278	0.7794	0.15
|NP_000430.3| proprotein convertase subtilisin/kexin type 1 preproprotein;	CN469887	3.7763	0.15
|NP_004244.1| phospholipase A2-activating protein; phospholipase A2 activating	BJ703212	0.0631	0.15
|NP_056271.2| cofactor of BRCA1; negative elongation factor protein B	BJ689040	0.0374	0.153
|NP_001545.2| cysteine-rich, angiogenic inducer, 61; cysteine-rich, anigogenic	BJ692405	0.0759	0.154
|NP_004073.2| dynactin 1 isoform 1; p150, Glued (Drosophila) homolog; dynactin 1	CN471126	0.0874	0.155
|NP_114072.1| frizzled 8; frizzled (Drosophila) homolog 8	DY626075	0.0422	0.157
|NP_542197.1| alpha 1 type XI collagen isoform C preproprotein; collagen XI,	BJ668630	0.035	0.159
|NP_078974.1| mitochondrial glutamate carrier 1	BJ688909	0.0905	0.162
|NP_005678.2| phenylalanine-tRNA synthetase-like, beta subunit; phenylalanyl-tRNA	BJ692171	0.0757	0.162
|NP_006197.1| platelet-derived growth factor receptor alpha precursor	BJ690723	0.0017	0.163
|NP_003240.1| thimet oligopeptidase 1	BJ691014	0.0778	0.164
|NP_009186.1| xylosylprotein beta 1,4-galactosyltransferase 7;	BJ695358	0.0745	0.165
|NP_872579.2| fetal Alzheimer antigen isoform 1; fetal Alz-50 reactive clone 1;	BJ701592	0.0349	0.165
|NP_057165.2| palladin; CGI-151 protein	BJ690747	0.0521	0.166
|NP_004521.1| matrix metalloproteinase 2 preproprotein; gelatinase neutrophil;	BJ694547	0.0645	0.166

|NP_056111.1| dedicator of cytokinesis 9	CN469939	0.0667	0.167
|NP_006651.2| ClpX caseinolytic protease X homolog; energy-dependent regulator of	BJ673221	0.0162	0.167
|NP_060004.2| myosin, heavy polypeptide 2, skeletal muscle, adult	BJ674015	0.0441	0.167
|NP_954580.1| ubiquitin-conjugating enzyme E2 variant 1	BJ685697	0.1036	0.168
|NP_149351.1| surfeit 4; surfeit locus protein 4; surface 4 integral membrane	BJ677148	0.0335	0.169
|NP_000503.1| N-acetylgalactosamine-6-sulfatase precursor; chondroitinase;	BJ693255	0.0526	0.169
|NP_001164.1| Rho GTPase activating protein 5	BJ686112	0.0363	0.172
|NP_003613.2| PTPRF interacting protein binding protein 1 isoform 1; liprin-beta 1;	BJ693077	0.0186	0.172
|NP_001449.1| gamma filamin; filamin C, gamma (actin-binding protein-280); filamin	BJ684635	0.063	0.173
|NP_001348.1| DEAH (Asp-Glu-Ala-His) box polypeptide 9 isoform 1; ATP-dependent RNA	DY630855	0.0189	0.174
|NP_000850.1| 3-hydroxy-3-methylglutaryl-Coenzyme A reductase	BJ700149	0.0228	0.176
|NP_631898.1| dipeptidylpeptidase 9; dipeptidyl peptidase 9; dipeptidyl peptidase	CN470065	0.0915	0.178
|NP_000628.1| glutathione reductase	BJ683618	0.0457	0.178
|NP_005955.1| myosin, heavy polypeptide 10, non-muscle; myosin heavy chain,	BJ696841	0.0186	0.179
|NP_932332.1| glucosamine-phosphate N-acetyltransferase 1	BJ691214	0.0418	0.182
|NP_002100.2| histidyl-tRNA synthetase; histidine-tRNA ligase; HisRS; histidine	BJ694216	0.0521	0.183
|NP_005782.1| M-phase phosphoprotein 10	DY628233	0.7037	0.184
|NP_065116.2| X-prolyl aminopeptidase (aminopeptidase P) 1, soluble; X-prolyl	BJ701510	0.0129	0.184
|NP_203750.3| mitogen-activated protein kinase 8 interacting protein 3 isoform 2;	CN468708	0.095	0.185
|NP_037457.3| APG4 autophagy 4 homolog B isoform a; autophagin-1	BJ693415	0.0586	0.185
|NP_002878.2| arginyl-tRNA synthetase	BJ697670	0.0023	0.186
|NP_006074.1| RED protein; RD element; prer protein; IK factor;	DY627427	0.0392	0.187
|NP_031401.1| TAR DNA binding protein; TAR DNA-binding protein-43	CN468754	0.0486	0.188
|NP_476500.1| endothelial differentiation, lysophosphatidic acid	DY630256	0.0536	0.188
|NP_003280.2| tropomyosin 2 (beta) isoform 1; arthrogryposis multiplex	BJ683045	0.0466	0.189
|NP_001339.1| death-associated protein kinase 3	BJ674422	0.0642	0.19
|NP_002145.3| heat shock 70kDa protein 4 isoform a; heat shock 70kD protein 4	BJ691037	0.0392	0.19
|NP_000180.2| hexokinase 2; hexokinase-2, muscle	BJ689488	0.0042	0.191
|NP_061957.2| cyclin J	BJ699157	0.0351	0.193
|XP_371474.2| PREDICTED: plexin B2	BJ684903	0.0552	0.195
|NP_004748.2| small inducible cytokine subfamily E, member 1; endothelial	BJ685307	0.0541	0.195
|NP_000149.1| glucan (1,4-alpha-), branching enzyme 1 (glycogen branching	BJ688578	0.0542	0.195
|NP_542164.2| oxysterol-binding protein-like 1A isoform B; oxysterol-binding	CN468818	0.0156	0.197
|NP_001027.2| ryanodine receptor 3	BJ694127	0.0848	0.198
|NP_000084.2| alpha 1 type V collagen preproprotein	BJ670714	0.1171	0.2
|NP_055316.1| ubiquitin carrier protein; ubiquitin-conjugating enzyme E2-24 kD;	BJ674752	0.0923	0.202
|NP_036322.2| formyltetrahydrofolate dehydrogenase isoform a	CN470753	0.2445	0.203
|NP_002787.2| proteasome beta 4 subunit; proteasome subunit, beta type, 4;	DY626588	0.1461	0.203
|NP_002513.1| neuronal pentraxin I precursor	CN468907	0.0914	0.204
|NP_057531.2| membrane protein, palmitoylated 6; MAGUK protein p55T; protein	CN469732	0.0431	0.204
|NP_075053.2| zinc transporter ZTL1; zinc transporter 5	BJ700354	0.0023	0.205
|NP_037428.2| G-protein signalling modulator 2 (AGS3-like, C. elegans); LGN	BJ695914	0.0205	0.207
|NP_000687.2| aldehyde dehydrogenase 9A1; gamma-aminobutyraldehyde dehydrogenase;	BJ701067	0.0726	0.207
|NP_056323.2| TCDD-inducible poly(ADP-ribose) polymerase	DY625879	0.0033	0.208
|NP_115984.1| protein phosphatase 1, regulatory subunit 9B; neurabin II;	BJ690319	0.0715	0.209
|NP_004664.1| angiopoietin-like 1 precursor; angiopoietin 3; angiopoietin Y1	BJ690922	0.0204	0.213
|NP_057375.1| inositol hexaphosphate kinase 2; mammalian inositol	DY631850	0.0558	0.213
|NP_001223.1| cardiac calsequestrin 2; calsequestrin 2, cardiac muscle;	BJ670795	0.0642	0.217
|NP_114402.1| N-myc downstream-regulated gene 3 isoform a; N-myc	CN470617	0.0521	0.219
|NP_002422.1| menage a trois 1 (CAK assembly factor); cyclin G1 interacting	DY628021	1.1881	0.221
|NP_055179.1| glyceraldehyde-3-phosphate dehydrogenase, spermatogenic;	BJ690842	0.1228	0.222
|NP_060729.2| tetratricopeptide repeat domain 17	DY627279	0.0152	0.222

|NP_775105.1| SNF2 histone linker PHD RING helicase; 2610103K11Rik	BJ697939	0.0346	0.222
|NP_006138.1| interferon regulatory factor 6; Popliteala pterygium syndrome	BJ685456	0.0388	0.223
|NP_002538.1| oligophrenin 1; oligophrenin-1, Rho-GTPase activating protein	BJ672227	0.064	0.224
|NP_057456.1| seven transmembrane domain orphan receptor; transmembrane domain	DY629566	0.0786	0.226
|NP_006789.1| UDP glycosyltransferase 2 family, polypeptide A1; UDP	DY629774	0.0155	0.228
|NP_116262.2| nm23-phosphorylated unknown substrate; SH3 domain-containing 70 kDa	BJ693084	0.0885	0.231
|NP_004206.1| estrogen receptor binding site associated antigen 9; cancer	BJ701417	0.0706	0.232
|NP_000184.1| sonic hedgehog preproprotein	BJ703040	0.0777	0.233
|NP_849189.1| sideroflexin 2	BJ687393	0.1028	0.233
|NP_110448.2| phospholipase A2, group XIIA; group XII secreted phospholipase A2;	BJ703348	0.0386	0.235
|NP_001783.2| cadherin 2, type 1 preproprotein; cadherin 2, N-cadherin	BJ680852	0.0642	0.237
|NP_071939.1| zinc finger, DHHC domain containing 6	DY628782	0.3895	0.239
|NP_055048.1| upstream binding transcription factor, RNA polymerase I	CN470191	0.0785	0.239
|NP_000144.1| galactosylceramidase precursor; galactocerebrosidase;	BJ676310	0.0262	0.24
|NP_000689.1| arachidonate 5-lipoxygenase	BJ698470	0.0846	0.242
|NP_006613.1| polo-like kinase 2; serum-inducible kinase	BJ697217	0.0071	0.243
|NP_003238.2| thrombospondin 2 precursor	BJ689989	0.05	0.244
|NP_060752.1| paraspeckle protein 1	CN468873	0.0041	0.244
|NP_001740.1| calpain, small subunit 1; calcium-activated neutral proteinase;	BJ670938	0.0626	0.245
|NP_056210.1| DKFZP434B0335 protein	BJ695451	0.4116	0.246
|NP_078932.2| hypothetical protein FLJ22329	BJ697568	0.2964	0.246
|NP_056349.1| zinc finger, ZZ domain containing 3	BJ688763	0.1254	0.247
